# Hybridization and introgression between toads with different sex chromosome systems

**DOI:** 10.1002/evl3.191

**Published:** 2020-08-19

**Authors:** Christophe Dufresnes, Spartak N Litvinchuk, Beata Rozenblut‐Kościsty, Nicolas Rodrigues, Nicolas Perrin, Pierre‐André Crochet, Daniel L Jeffries

**Affiliations:** ^1^ LASER College of Biology and the Environment Nanjing Forestry University Nanjing People's Republic of China; ^2^ Department of Animal and Plant Sciences University of Sheffield Sheffield United Kingdom; ^3^ Institute of Cytology Russian Academy of Sciences Saint Petersburg Russia; ^4^ Dagestan State University Makhachkala Russia; ^5^ Department of Evolutionary Biology and Conservation of Vertebrates Faculty of Biological Sciences University of Wrocław Wrocław Poland; ^6^ Department of Ecology & Evolution University of Lausanne Lausanne Switzerland; ^7^ CEFE Univ. Montpellier, CNRS, EPHE, IRD Univ Paul Valéry Montpellier 3 Montpellier France

**Keywords:** *Bufo bufo*, *Bufo spinosus*, hybrid zone, RADseq, reproductive isolation, sex chromosome turnover, speciation

## Abstract

The growing interest in the lability of sex determination in non‐model vertebrates such as amphibians and fishes has revealed high rates of sex chromosome turnovers among closely related species of the same clade. Can such lineages hybridize and admix with different sex‐determining systems, or could the changes have precipitated their speciation? We addressed these questions in incipient species of toads (Bufonidae), where we identified a heterogametic transition and characterized their hybrid zone with genome‐wide markers (RADseq). Adult and sibship data confirmed that the common toad *B. bufo* is female heterogametic (ZW), while its sister species the spined toad *B. spinosus* is male heterogametic (XY). Analysis of a fine scale transect across their parapatric ranges in southeastern France unveiled a narrow tension zone (∼10 km), with asymmetric mitochondrial and nuclear admixture over hundreds of kilometers southward and northward, respectively. The geographic extent of introgression is consistent with an expansion of *B. spinosus* across *B. bufo*’s former ranges in Mediterranean France, as also suggested by species distribution models. However, widespread cyto‐nuclear discordances (*B. spinosus* backrosses carrying *B. bufo* mtDNA) run against predictions from the dominance effects of Haldane's rule, perhaps because Y and W heterogametologs are not degenerated. Common and spined toads can thus successfully cross‐breed despite fundamental differences in their sex determination mechanisms, but remain partially separated by reproductive barriers. Whether and how the interactions of their XY and ZW genes contribute to these barriers shall provide novel insights on the debated role of labile sex chromosomes in speciation.

**Impact Summary**

In mammals, sex is determined by a pair of XX/XY sex chromosomes: the Y chromosome triggers male development, hence males are XY and females are XX. This is the reverse in birds, where females carry the heterogametolog (noted W); females are ZW and males are ZZ. Because the Y and W are transmitted clonally and degenerate, sex chromosomes usually evolve faster than the rest of the genome, and eventually contribute genetic incompatibilities between diverging lineages. However, in other vertebrates such as amphibians and fishes, sex chromosomes are reshuffled frequently and closely related lineages may differ in their mechanisms of sex determination. Can they still hybridize and mix their genomes with different sex chromosomes? We addressed this question in two toad taxa, *Bufo bufo*, and *Bufo spinosus*. By screening thousands of genetic markers across males and females, we first showed that *B. bufo* is ZW while *B. spinosus* is XY, evidence that one species experienced a shift of sex chromosomes since their initial divergence several million years ago. We then accurately characterized their hybrid zone in southeastern France with hundreds of species‐diagnostic markers along a densely sampled transect. Despite their different sex‐determining systems, the two species can successfully hybridize, but only across a narrow transition (10km wide) where reproductive isolation (genetic incompatibilities) likely prevent their gene pools from merging. Parts of the genome diffused hundreds of kilometers inside species ranges, which is consistent with a northern expansion of the southern species (*B. spinosus*) into the range of its counterpart (*B. bufo*). Importantly, we report asymmetric patterns of admixture between the nuclear compared to the maternally inherited mitochondrial DNA, which could result from uneven sex ratio in hybrids of varying gametolog composition (XW, YW, XZ, YZ). Our study suggests that the evolution of different sex chromosomes did not seal reproductive barriers between these toads and offer a promising framework to understand whether and how the interactions of their XY and ZW genes contribute to their speciation.

Almost a century after the conception of Haldane's rule (Haldane 1922), the role played by sex chromosomes in speciation remains an exciting topic for evolutionary biologists (Payseur et al. 2018). Because they usually evolve faster (due to less efficient purifying selection and/or enhanced adaptation), bear more genes, and are hemizygous in the heterogametic sex (XY males and ZW females), X and Z chromosomes are expected to disproportionally contribute to genetic incompatibilities underlying post‐zygotic isolation (hybrid unviability and sterility), in comparison to the rest of the genome (Qvarnström and Bailey 2009; Schilthuizen et al. 2011; Beukeboom and Perrin 2014). Empirical evidence for this effect is readily observed in mammals (Payseur et al. 2004), birds (Storchová et al. 2010), and *Drosophila* (Presgraves 2008), where sex determination is stable and sex chromosomes have been decaying for tens/hundreds of million years (Beukeboom and Perrin 2014). In contrast, the effect is more elusive in taxa with young, less stable sex chromosomes (Lima 2014). Hybrid incompatibilities may quickly accumulate on recently evolved Y chromosomes in the early stages of differentiation (Dufresnes et al. 2016; Hu and Filatov 2016; Filatov 2018), but remain essentially autosomal when recombination prevents X–Y divergence (Macaya‐Sanz et al. 2011; Gerchen et al. 2018). Taxonomic groups that exhibit a high diversity of sex chromosomes, with both male (XY) and female (ZW) heterogamety, thus hold the key to understanding how sex‐linked genes affect reproductive isolation and speciation (Filatov 2018; Ogata et al. 2018).

Due to the lability of sex determination in fishes (Kitano and Peichel 2012) and amphibians (Miura 2017), closely related lineages sometimes possess different sex chromosome systems. What happens when these systems meet in secondary contact? Given the importance of sex‐linked genes in hybrid incompatibilities (Qvarnström and Bailey 2009), carrying non‐homologous sex chromosomes should generally increase reproductive isolation and thus the probability to speciate. For instance, genic conflicts could drastically alter sex determination and gametogenesis in hybrids, causing intersex or sterile individuals, respectively, in turn building strong post‐zygotic barriers. Combining independently evolved sex chromosomes could also promote new combinations of sexual characters. In sticklebacks, the evolution of neo‐sex chromosomes has driven inter‐species phenotypic divergence, which triggered speciation events (Kitano et al. 2009; Kitano and Peichel 2012). Alternatively, the systems may remain fully compatible (e.g., if one supersedes the other), resulting in porous species boundaries and admixed populations, as documented in the Japanese wrinkled frog *Glandirana rugosa* (Miura et al. 1998; Ogata et al., 2003; Miura 2007,2018). These opposite outcomes illustrate the difficulty to predict the consequences of colliding sex‐determining systems on the speciation process, which so far have been studied only in a handful of cases.

We focused on these fascinating aspects in two incipient species of toads, *Bufo bufo* and *B. spinosus*. These widespread amphibians form a 900‐km‐long transition across France, which has been extensively characterized using morphology, mitochondrial DNA (mtDNA) and various sets of nuclear markers (Arntzen et al., 2016, 2017, 2018; van Riemsdijk et al. 2019). Previous studies uncovered puzzling patterns of genetic diversity and introgression. On the one hand, analyses of local transects quantified abrupt shifts in allele frequencies that may be mediated by reproductive isolation and/or demographic processes such as hybrid zone movement (Arntzen et al. 2016; van Riemsdijk et al. 2019). On the other hand, the widespread presence of “*bufo*” mtDNA and allozyme alleles among southern populations (assigned to *B. spinosus*), hundreds of kilometers away from the identified contact zones, has been interpreted as a sign of widespread admixture, initially blurring their validity as distinct species (García‐Porta et al. 2012, but see Recuero et al. 2012 and Arntzen et al. 2013). Arntzen et al. (2017) tentatively reconciled these conflicting observations under a biogeographic scenario involving multiple spatial shifts of the hybrid zone, following the successive expansions of each species from their respective Iberian (*B. spinosus*), and Alpine/Balkan glacial refugia (*B. bufo*) since the last ice age. But because previous phylogeographic studies were limited to mtDNA and a few introns (Arntzen et al. 2017) or allozymes (García‐Porta et al. 2012), it remains unclear whether “foreign” alleles genotyped as far as the Mediterranean coast and the Jura Mountains reflect past introgression or shared ancestral polymorphism. The high genetic resolution now offered by high‐throughput genomic approaches has the potential to distinguish among these hypotheses and better understand the factors that mediate admixture at this iconic hybrid zone.

Among potential incompatibilities, *B. bufo* and *B. spinosus* may exhibit fundamental differences in their sex‐determining systems. Common toads from the *B. bufo* complex were proposed to be female‐heterogametic (ZW) early on (Ponse 1942), which was for long considered the rule among bufonids (e.g., Malone and Fontenot 2008). Nevertheless, sex chromosome turnovers and male‐heterogamety do occur in this family (Stöck et al. 2011). In *Bufo*, the experiments of Ponse (1950) and Rostand (1952,1953) suggested contradictory patterns of heterogamety depending on the geographic origins of specimens. Recently, Skorinov et al. (2018) characterized a karyotypic dimorphism specific to males in *B. spinosus*, based on a few individuals. Hence, *B. bufo* and *B. spinosus* could differ in their heterogametic sex chromosomes (ZW versus XY), which in turn would allow testing whether such a recent turnover have affected their propensity to admix at range margins, and precipitated their incipient speciation.

In this study, we revisit the *B. bufo*/*spinosus* hybrid zone with genome‐wide data (RADseq), specifically to (i) confirm a putative heterogametic transition of sex‐determining systems between the two species and (ii) illuminate their patterns of admixture along an extensive transect in southeastern France. We predict substantial introgression mediated by geographic and demographic processes if the different sex‐determining systems between the two species remained compatible and did not seal their reproductive barriers.

## Methods

### FIELD SAMPLING

A total of 576 individuals of common and spined toads were captured in February–April 2016 from 67 localities between French Catalonia and Western Switzerland, broadly covering the *B. bufo*/*spinosus* transition (Table S1). Most individuals were live adults (*n* = 525), subsequently released after DNA collection with non‐invasive buccal swabs. Additional tissue samples were also obtained from road kills (*n* = 21) and small larvae (*n* = 30), fixed in 70–96% ethanol. To find sex‐linked markers, our sampling scheme involved an extensive set of males and females (*n* ≥ 20 of each sex) from two localities far from the contact zone, and confirmed as pure *B. spinosus* (loc. 3; Aumelas, France: 43.5743°N, 3.6442°E) and pure *B. bufo* (loc. 21; Aubonne, Switzerland: 46.5083°N, 6.3691E). Beyond general characteristics (size, color, body shape), sex was diagnosed by the presence (males) or absence (females) of nuptial pads on the first fingers, a dimorphism particularly marked during the breeding season (Dufresnes 2019).

In order to get additional evidence for the sex‐determining system of *B. bufo*, we also analyzed phenotypically sexed offspring obtained from a controlled cross—sex‐specific polymorphism is expected to be more readily detectable among sibs than randomly sampled adults of various ancestry. One mating pair (amplexus) was captured at loc. 21 and kept in a large container (525 L) until spawning (a few days), after which both parents were released in their place of capture. Tadpoles were raised until metamorphosis and a subset of 60 froglets (Gosner stage > 42–45) was euthanized by overdose of MS222 (0.15 g/L, buffered with sodium bicarbonate 0.3g/L) and fixed in 70% ethanol. Tissue samples (hindlegs) were collected for downstream genetic analyses. Froglets were then sexed by dissection and histological analysis of their gonads (Fig. S1). Gonads were first post‐fixed for 2 h in Bouin's solution (Sigma) and rinsed in 70% ethanol. The gonads were photographed using a cooled Carl Zeiss Axio‐Cam HRc CCD camera mounted on a Stemi SV11 (Zeiss) microscope. Afterward, gonads were dehydrated, embedded in paraplast, sectioned into 7‐μm slices, stained with Mallory's trichrome, and staged according to Ogielska and Kotusz (2004) and Haczkiewicz and Ogielska (2013).

### LABWORK

The DNA of all samples was extracted using the Qiagen Biosprint robotic workstation. Four separate genomic libraries were prepared following the double‐digest RADseq protocol fully described in Brelsford et al. (2016a). Briefly, this consists of enzyme digestion (here using *SbfI* and *MseI*), ligation of individual barcodes (on the *SbfI* end), amplification of the ligated fragments, and size selection between 400 and 500 bp. Library 1 included 41 adult male and female reference samples of *B. bufo*. Library 2 included 40 adult male and female reference samples of *B. spinosus*. Library 3 included 35 *B. bufo* metamorphs that could be confidently sexed. Bufonid samples from other projects completed these libraries (up to 96 samples) and each library was sequenced on two Illumina HiSeq 2500 lanes (single read 125). Library 4 included 200 toad samples collected from 21 localities across the hybrid zone (Table S1) and was sequenced on three Illumina lanes (single read 125). Raw reads were quality‐checked and filtered with FastQC version 0.10.1 (Andrews 2010), and demultiplexed with Stacks version 1.48 (Catchen et al. 2013).

The majority of wild‐caught toads (*n* = 514) were mitotyped by sequencing a short fragment (∼500 bp) of the gene *cytochrome‐b* (*cyt‐b*), amplified with the following custom primers: CytB.Bufo.Mid.F (5’–ATTATTGCAGGCGCCTCAATA–3’) and CytB.Bufo.R (5’–AGTTTRTTTTCTGTGAGTCC–3’). PCRs were carried out in 25 μL reactions containing 3 μL of template DNA, 7.5 μL of multiplex master mix (Qiagen, containing buffer, dNTPs and hot‐start polymerase), 1 μL of each primer (10 μM), and were run as follows: 95°C for 15’; 35 cycles of 94°C for 30”, 53°C for 45” and 72°C for 1’; 72°C for 5’. Sequences were manually aligned in Seaview (Gouy et al. 2010) and matched against reference haplotypes from the two species (GenBank sequences from Recuero et al. 2012).

Finally, we further incorporated the average mitochondrial and nuclear allele frequencies of *B. bufo*/*spinosus* obtained for ∼200 populations of southeastern France and nearby Italy by Arntzen et al. (2017, taken from their tables S1 and S4, respectively), for complementation and comparison.

### SCREENING FOR SEX‐LINKED LOCI

Sexed adults and siblings of *B. bufo* (libraries 1 and 3), as well as adults of *B. spinosus* (library 2), were screened for putative sex‐linked markers, applying two independent approaches. The first approach, SLM finder, is an ad hoc pipeline developed to identify SNPs that show sex biases in allele frequencies (method I), heterozygosity (method II), or RADseq tags that are specific to only one sex (method III) (Breslford et al. 2016b; Jeffries et al. 2018). For this, loci were constructed and SNPs were called using Stacks version 1.48. Default parameters were used for the *Ustacks* and *Cstacks* modules, as these represented the best balance between stringency and data inclusion. For the *Populations* module, SNP genotypes were retained if the locus coverage was at least 8 reads (*–m* 8), if called for ≥80% of individuals of each sex (*–r* 0.8, *–p* 2) and if heterozygosity across the whole dataset was not greater than 75% (*–max_obs_het* 0.75), to remove over merged paralogous loci.

In order to setup the optimal parameters with which to identify sex‐linked markers, we ran SLM finder for 24 different combinations of the parameters underlying methods I and II (heterogametic and homogametic thresholds), and for six parameter values of method III (sex specificity threshold; Table S2). For each set, we performed a permutation test whereby male and female sex assignments were randomly shuffled across the entire sample set 100 times, SLM finder was run, and the number of putative sex‐linked markers obtained was recorded. This null distribution was then compared to the number of sex‐linked markers flagged on the observed data (i.e., real sex assignments). If this number fell above the 95th percentile of the null distribution, we considered sex linkage to be significant (*P* < 0.05). For a given method, we could further select the optimal parameter set as the one minimizing false positives, that is, showing the smallest proportion of sex‐linked markers in the null distribution relative to the number of sex‐linked markers in the observed data.

The second approach, RadSex (https://github.com/RomainFeron/RadSex), identifies sex specific RADseq tags or alleles directly from the raw read data (i.e., without locus assembly or SNP calling), and compare their presence among samples of each sex. This approach has the advantage that it does not require the assembly of loci, which has two important consequences: (i) it is sensitive to only one parameter, namely the coverage of unique RAD tags (*–min_cov*), hence reducing the potential for parameter biases; (ii) complex loci that are usually discarded by assembling pipelines such as Stacks (e.g., indel or polyallelic markers) are considered in the analysis. The coverage table was created (*radsex process*) using a minimum coverage of 1 read (*–min_cov* 1). The distribution table, which summarizes the number of males and females in which each unique sequence is present (*radsex distrib*) was created using a minimum coverage of 5 reads (*–min‐cov* 5). These values allow to retain low coverage loci while removing reads that contain potential sequencing errors (present in single copies only) or PCR errors (present in several copies only). Markers showing statistically significant association with sex were identified via Chi‐squared test using Yate's correction for continuity and Bonferroni correction for multiple testing.

### HYBRID ZONE ANALYSES

The 200 hybrid zone samples (library 4) were processed with the *denovo.pl* pipeline of Stacks. Default stacking parameters were again used for *Ustacks* and *Cstacks* modules. In a first step, we filtered for SNPs genotyped in at least 90% of the samples (–*r* 0.9) in all localities (–*p* 21), which yielded 8,560 SNPs (99.3% of matrix completeness). The genetic structure of this initial dataset was explored by a Principal Component Analysis (PCA) on individual genotypes with the R package *adegenet* (Jombart 2008). For specific hybrid zone analyses, we then called species‐diagnostic SNPs, as those fixed between populations at the edges of our study area, specifically, between loc. 1–3 (*B. spinosus*) and loc. 20–21 (*B. bufo*), retaining only one SNP per RADseq tag, which yielded 950 SNPs. We performed a PCA as above, and estimated the ancestry of individuals to either species with the Bayesian clustering algorithm of STRUCTURE (Pritchard et al. 2000), focusing on runs with *K* = 2. We applied the admixture model without pre‐assignment of samples, and performed 10 replicate chains of 100,000 iterations, after a burn‐in of 10,000. Replicates were combined with CLUMPP (Jakobsson and Rosenberg 2007). For each population, we computed observed heterozygosity (*H_o_*) with the R package *hierfstat* (Goudet 2005), linkage disequilibrium (correlation coefficient *R^2^*) averaged over all pairs of loci with the R package *genetics* (Warnes et al. 2013), and the admixture linkage disequilibrium (*D’*) as the variance in individual hybrid index (Barton and Gale 1993), here using the genome‐wide individual ancestry obtained with STRUCTURE (individual *Q* scores) as a proxy. *H_o_* was also computed for each individual.

The hybrid zone was further characterized by fitting sigmoid clines to allele frequency changes along the northeast‐southwest geographic transect covered by our sampling, using the *R* package *hzar* (Derryberry et al. 2014). We first computed clines for mtDNA and the average nuclear ancestry obtained with STRUCTURE (population *Q* scores), and performed model selection (AIC) on clines with two (center *c* and width *w*) to six parameters (length *δ* and slope *τ* of the exponential tails). Maximum (*P_max_*) and minimum (*P_min_*) allele frequencies were set to 0 and 1, respectively, since the considered markers/indices were fixed between our edge populations. Second, to explore the heterogeneity of introgression throughout the genome, we fitted clines to each of the 950 SNPs individually, using models with six parameters for comparability. We specifically compared the tail parameters δ and τ to quantify whether introgression was asymmetric, which should be reflected by longer and steeper tails on one side of the cline compared to the other one (Barton and Gale 1993).

### SPECIES DISTRIBUTION MODELING

To grasp whether environmental factors (adaptation to different ecological niches) contributed to mediate the transition between *B. bufo* and *B. spinosus*, we built species distribution models. We applied recent methodological recommendations to compute robust ecological niche models with MaxEnt 3.4.1 (Phillips et al. 2006), such as occurrence filtering, using multiple combinations of model parameters (features and regularization multipliers), and multiple statistical criteria for model selection (partial ROC, omission rates, and AICc).

A total of 14,504 localities known of *B. bufo* and *B. spinosus* were initially considered, combining our own records, museum collections, and previously published data. We first filtered this dataset to avoid spatial autocorrelation and duplication using NicheToolBox (Osorio‐Olvera et al. 2018). We then retained the localities at least 10 km (0.093°) apart (see Brown 2014), and the final dataset comprised 3212 records for *B. bufo* and 886 records for *B. spinosus* (see Fig. S2).

To compute the models, altitude and 19 bioclimatic layers representative of the climatic data over ∼1950–2000 were extracted from the WorldClim 1.4 database (http://www.worldclim.org). Ten additional layers were considered: the aridity index (Global Aridity and Potential Evapo‐Transpiration; http://www.cgiar-csi.org/data/global-aridity-and-pet-database), the global percent of tree coverage (https://github.com/globalmaps/gm_ve_v1), and eight land cover variables (spatial homogeneity of global habitat, broadleaf forests, needleleaf forests, mixed forests, shrubs, barren, herbaceous, and cultivated vegetation; https://www.earthenv.org/). To also consider topography in the model, four landscape layers were calculated with QGIS: aspect, exposition, slope, and terrain roughness index. These layers had a 30 arc seconds spatial resolution. All analyses were conducted under the WGS 84 projection with species‐specific masks covering the areas of occurrence of *B. bufo* and *B. spinosus*.

To eliminate predictor collinearity before generating these models, we calculated Pearsons's correlation coefficients for all pairs of bioclimatic variables using ENMTools (Warren et al. 2010). For correlated pairs (│r│> 0.75), the variable that appeared the most biologically important for the species was retained. The resulting dataset contained ten bioclimatic variables for *B. bufo*: Bio 1 (annual mean temperature; °C × 10), Bio 2 (mean diurnal range; °C × 10), Bio 3 (isothermality; Bio2/Bio7 × 100), Bio7 (temperature annual range; °C × 10), Bio 8 (mean temperature of wettest quarter; °C × 10), Bio 14 (precipitation of driest month; mm), Bio 15 (precipitation seasonality; CV), Bio 16 (precipitation of wettest quarter; mm), Bio 18 (precipitation of warmest quarter; mm), and Bio 19 (precipitation of coldest quarter; mm); and five bioclimatic variables for *B. spinosus*: Bio 3, Bio 6 (minimum temperature of coldest month; °C × 10), Bio 8, Bio 14, and Bio 16. We then applied a jackknife analysis to estimate the relative contributions of variables to the MaxEnt model.

MaxEnt models were ran with 10 replicates. Model calibration consisted in the evaluation of models created with distinct regularization multipliers (0.5 to 6, at intervals of 0.5) and feature classes (resulted from all combinations of linear, quadratic, product, threshold, and hinge response types). The sets of variables consisted of 25 layers for *B. bufo* and 20 for *B. spinosus*. The best parameter settings were selected considering statistical significance (partial ROC), predictive power (omission rates E = 5%), and complexity level (AICc), obtained using the *R* package kuenm (Cobos et al. 2019).

To assess whether the parapatric ranges correspond to an environmental transition for the species, we examined how their occurrence probability varied among the sampled populations of our transect. In addition, we performed multivariate analyses (PCA, R package *ade4*) on the geoclimatic variables at occurrence records, contrasting those from France (where the transition is located) and the rest of the species ranges.

## Results

### SEX‐LINKED MARKERS

Testing different parameter combination using a permutation approach allowed to set up the optimal parameters set of SLM finder, which were used to identify putative sex‐linked markers from the adult and sibling datasets of *B. bufo*, as well as the adult dataset of *B. spinosus* (Fig. S3 and Table S2).

**Figure 1:**
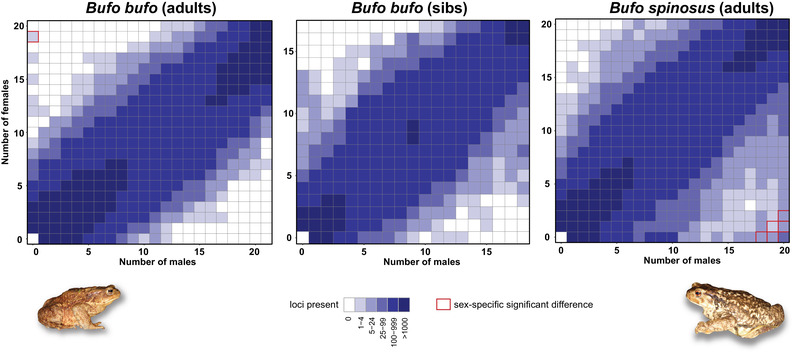
Sex‐specific presence of RAD variants in *B. bufo* (from the adult and sib datasets) and *B. spinosus* (adults only) as inferred by the RADsex approach. Differences reaching statistical significance are squared in red. In *B. bufo*, the analysis counted more tags specific to females (vertical axis) than males (horizontal axis), but a single one in significant proportions. In *B. spinosus*, tens of tags are significantly specific to males.

For *B. bufo*, the adult dataset comprised 146,292,576, reads after demultiplexing, which contained 35,084 SNPs after the Stacks processing (average coverage = 32.3 reads/locus). No support was found for either XY or ZW patterns of sex‐linkage in this dataset, regardless of the parameter combination (Table 1). In contrast, the RadSex approach highlighted one significantly sex‐linked marker consistent with a ZW system, that is, a sequence found among most of the females (19/20), but none of the males (0/21; Fig. 1, left panel). This sequence contains an indel polymorphism and was therefore discarded by the Stacks pipeline, which is why it was not present (and flagged) among the loci screened by SLM finder.

**Table 1:** Number of SNPs consistent with a XY and a ZW system in *B. bufo* (adults: 21 males and 20 females; sibs: 18 males and 17 females) and *B. spinosus* (adults: 20 of each sex) identified by SLM Finder under the allele frequency (I), heterozygosity (II) and tag dropout (III) approaches. Higher numbers than expected by chance (*P* ≤ 0.05) are highlighted by asterisks, according to permutation tests (Fig. S3)

	*B. bufo*			
	I	II	III	Total
Adults (23,007 loci)				
XY	0	0	0	0
ZW	0	0	0	0
Sibs (11,465 loci)				
XY	0	1	0	1
ZW	16*	15*	0	16

	*B. spinosus*			
	I	II	III	Total
Adults (28,089 loci)				
XY	90*	89*	2	104
ZW	2	1	1	4

The *B. bufo* sibling dataset comprised 122,605,407 reads after demultiplexing, which contained 12,840 SNPs after the Stacks processing (average coverage = 39.1 reads/locus). SLM finder tests also found evidence for a ZW system from this data. With the optimal thresholds, the frequency and heterozygosity methods of SLM finder reported 16 and 15 putative ZW loci, respectively (15 supported by both methods; Table 1). Only one putative XY marker was identified (by the heterozygosity method), which fell within the expected rate of false positive (Fig. S3). Note that for the heterozygosity method, more relaxed parameters on the homogametic threshold yielded a significant male‐heterogametic signal (Fig. S3). This could reflect Z‐specific polymorphisms between the father's Zs (ZZ) and the mother's Z (ZW), hence mimicking an XY‐like allele segregation in the full‐sib clutch, as demonstrated for similar datasets (e.g., *Rana montezumae*, Jefrries et al. 2018). RadSex also suggested a ZW system among the sibling dataset: many tags were sequenced exclusively among the females (11–13/17), although the bias was not significant (Fig. 1, central panel).

The *B. spinosus* adult dataset comprised 452,840,434 reads after demultiplexing, which contained 28,090 SNPs (average coverage = 37.2 reads/locus). SLM finder found strong evidence for an XY system (Table 1). Altogether, a total of 104 XY loci were flagged, 89 of them being detected by both the allele frequency and the heterozygosity methods, and in highly significant numbers (Fig. S3b). In contrast, only four tags fit a ZW pattern, which was within random expectations. The RadSex results also recovered an XY system, with 151 significantly male‐specific RAD tags, but no female‐specific ones (Fig. 1, right panel). About a third (48 markers) corresponds to those recovered by SLM finder, while the rest were discarded by the Stacks pipeline (see Methods).

### HYBRID ZONE ANALYSES

**Figure 2:**
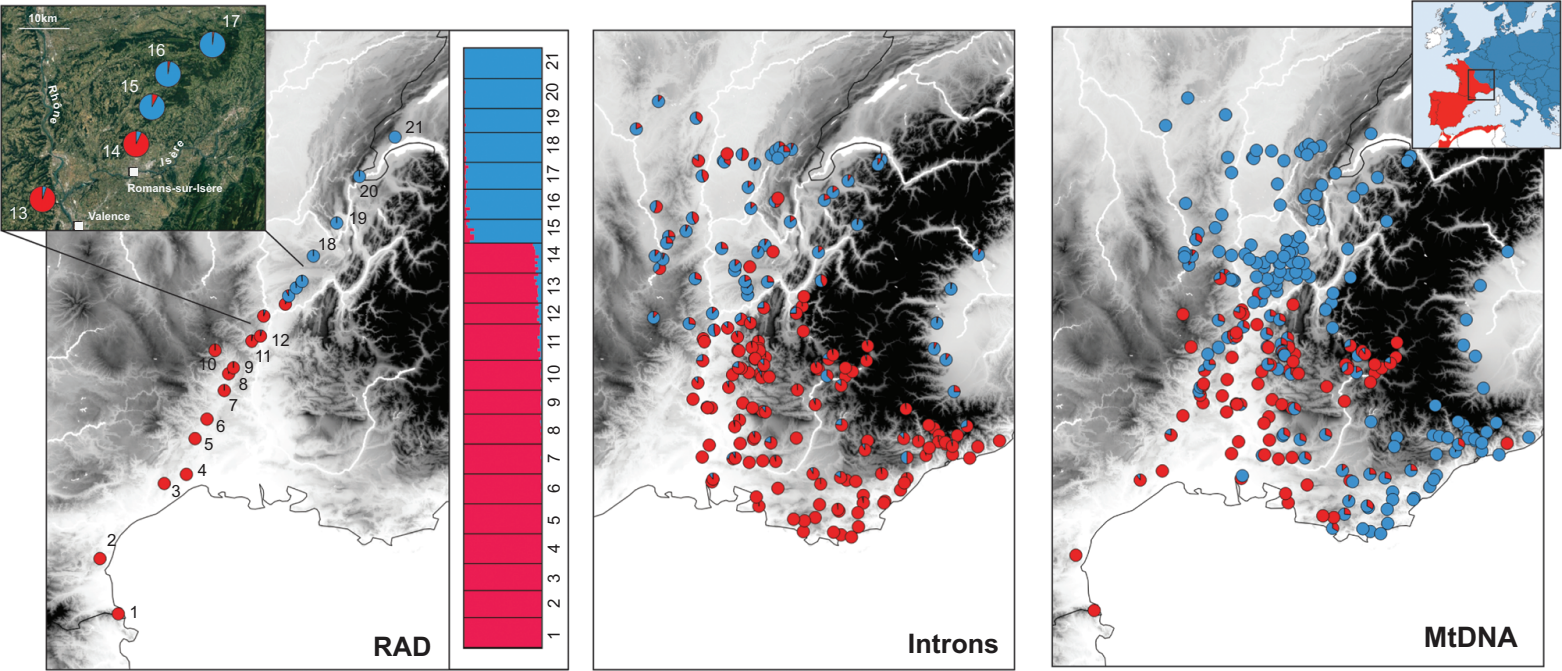
The hybrid zone between *B. bufo* (blue) and *B. spinosus* (red) in southern France. The left panel presents our nuclear clustering analyses based on RADseq data (950 species‐diagnostic loci, i. e. fixed between edge populations); the left inset map zooms in the area of contact. The middle panel reports population ancestry obtained from the analysis of intron markers BDNF, POMC, RAG1 and RPL3 (Arntzen et al. 2017); many individuals with *B. spinosus* or intermediate ancestries at these loci were sampled in ranges that clearly belong to *B. bufo* (according to the RADseq data). The right panel combines mitochondrial barcoding from ours and Arntzen et al. (2017). The top right inset map shows the distribution of the two species in Western Europe (adapted from Dufresnes 2019).

The distributions of the *B. bufo* and *B. spinosus* genomes in southeastern France are displayed in Fig. 2, according to RADseq, introns and mitochondrial markers. Our initial RADseq dataset (8,560 SNPs) recovered the two gene pools as the main source of genetic structure, which built the first PCA component (PC1 in Fig. S4). It also highlighted some intraspecific structure: the southernmost *B. spinosus* samples (loc. 1–2), and the northernmost *B. bufo* samples (loc. 20–21) form the second and third PCA components, respectively (Fig. S4).

**Figure 3:**
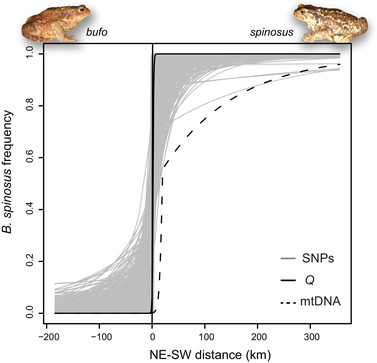
Geographic clines fitted to the proportion of mitochondrial DNA, nuclear genome average (STRUCTURE *Q*), and allele frequency at 950 species‐diagnostic SNPs (i.e., fixed between edge populations) along our *B. bufo*/*spinosus* transect running from Switzerland (NE) to French Catalonia (SW). Distances are given as the deviation from the median center.

As expected, the 950 SNPs fixed between the groups of populations at the edges of our transect (loc. 1–3 and loc. 20–21) mostly captured the genetic differentiation between the two species (63.8% of variance explained by PC1 in Fig. S4). The STRUCTURE ancestry coefficients obtained from this dataset closely matched the PC1 scores, indicative of robust inferences (Fig. S5). The clustering analysis supported a sharp nuclear transition between our loc. 14 and 15, located just 9 km apart in the hills north of Romans‐sur‐Isère (Fig. 2), associated with an increase of heterozygosity (*H_o_*) and linkage disequilibrium (Fig. S6). Fainted traces of admixture were detected up to a hundred kilometers in either direction (loc. 7–14 and loc. 15–20, Fig. 2), but all ancestry coefficients reached above 0.9, even close to the putative center (Fig. 2, Fig. S7). Accordingly, the cline fitted to the average population ancestry (STRUCTURE's *Q*) along our transect was only 8.6 km wide, with the best model involving mirrored introgression tails on both sides (Fig. 3 and Table 2). The geographic transition inferred from nuclear introns (Arntzen et al. 2017) is comparatively more variable, with genotypes assigned to *B. spinosus* found among populations of *B. bufo* (Fig. 2).

**Table 2:** Summary of the cline analyses. For the mtDNA and nuclear *Q* scores, parameter estimates for *w* and *c* are provided with their 95% confidence interval. For the 950 species‐diagnostic SNPs, the median and the 95% distribution of parameters are shown

	mtDNA	Nuclear (*Q*)	Nuclear (SNPs)
Best model	one tail (*B. spinosus*)	mirrored tails	–
Model likelihood	−24.6	−1.3	–
Width *w*	11.4 (3.4‐35.1)	8.6 (8.6‐14.8)	11.7 (2.3–64.6)
Center *c*	203.6 (194.6–209.6)	185.9 (183.5–186.3)	184.1 (179.5–191.2)

The mitochondrial transition was less clear cut: *B. bufo* mitotypes are widespread south of the nuclear transition, and even far within the range of *B. spinosus*, while no *B. spinosus* mtDNA was detected among *B. bufo* populations (Fig. 2). As a result, the best model for the mitochondrial cline involved an introgression tail on the *B. spinosus* side, a narrow width (11.4 km), and a center shifted about 20 km south compared to the nuclear cline (around Valence, near loc. 13; Figs. 2–3 and Table 2).

The clines fitted independently to each of the 950 species‐diagnostic loci are shown in Fig. 3. The width and center estimates followed unimodal distributions with medians of 11.7 and 184.1 km (equidistant to loc. 14 and 15), respectively (Table 2; Fig. S8). Introgression tails were significantly longer (higher *δ*) and steeper (higher *τ*) on the *B. bufo* than on the *B. spinosus* side (*P* ⋘ 0.05 for *δ* and *P* = 0.04 for *τ*; paired Wilcoxon signed ranked tests; Fig. S9).

### SPECIES DISTRIBUTION MODELING

A total of 348 replicate models for each species were assessed for calibration, all of which were statistically significant when compared with a null model of random prediction. Of these significant models for *B. bufo*, a single model significantly met both the omission criterion of 5% and the AICc criteria (Table S3). Mean diurnal range (16%), broadleaf forest (16%), cultivated vegetation (15%), tree coverage (15%), and shrubs percent (13%) altogether contributed 75% of the model (Table S3). For *B. spinosus* no model met the omission criterion and a single model significantly met the AICc criteria (Table S3). Of the parameters included in the model, cultivated vegetation (38%), tree coverage (17%), mixed forest percent (13%), and mean temperature of wettest quarter (12%) altogether contributed 80% (Table S3).

**Figure 4:**
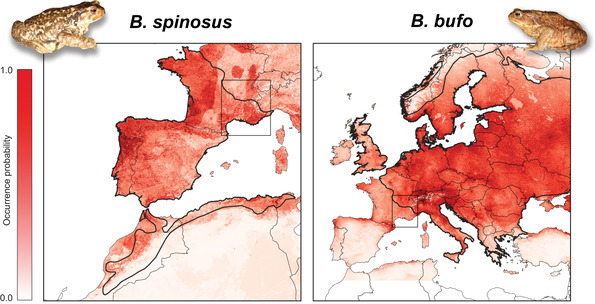
Projected distributions of *B. bufo* and *B. spinosus* according to their bioclimatic models. Thick lines show range limits. The area of contact studied here (frame) is suitable for both species, particularly along the Rhône valley for *B. spinosus*.

The projected distributions of *B. bufo* and *B. spinosus* under the selected models are displayed in Fig. 4. For both species, large parts of France received intermediate occurrence probabilities, but most records fall within the edges of the geoclimatic parameter space of both species, supporting that these areas correspond to some environmental‐ecological boundaries (Fig. S10). This ecological transition was steeper for *B. bufo* than for *B. spinosus*: along our transect, the suitability for *B. bufo* significantly decreased with geographic distance, while the relationship was not significant for *B. spinosus* (Fig. S11).

## Discussion

Can the evolution of distinct sex‐determining systems precipitate speciation events between diverging lineages? Our study shows that the heterogametic transition in the toads *B. bufo* (ZW) and *B. spinosus* (XY) was insufficient to prevent admixture, although their narrow hybrid zone implies some post‐zygotic isolation.

This recent turnover—the two species are 5–10 million years old (García‐Porta et al. 2012, Recuero et al. 2012)—bolsters the emerging view that sex chromosomes evolve rapidly within amphibian radiations, as seen from large‐scale studies in Ranid (Jeffries et al. 2018) and Hylid frogs (Dufresnes et al. 2015). In other bufonids, female heterogamety has been reported in the genus *Rhinella* (Abramyan et al. 2009), while *Bufotes* (Stöck et al. 2011), *Strauchbufo* (Deng and Shang 1984), and *Duttaphrynus* (Siripyasing et al. 2008) are male‐heterogametic. Female heterogamety is probably the ancestral state in *Bufo*, since ZW chromosomes were also characterized in *B. gargarizans* (Wen et al. 1983), a close Asian relative of *B. bufo* and *B. spinosus*. These recurrent heterogametic switches are rather uncommon: transitions are expected to preserve the heterogametic sex, due to mutation load selection and drift (Blaser et al. 2014; Saunders et al. 2018). In amphibians, phenotypic males recombine far less than females (Bufonids included, Stöck et al. 2013), so a Y should differentiate faster from the X than the W from the Z. Accordingly, we found much larger numbers of XY markers in *B. spinosus* than of ZW markers in *B. bufo*, despite stronger resources for the latter species. This also makes homomorphic ZW chromosomes more difficult to detect, which could contribute to why male heterogamety appear more prevalent in some amphibian groups (Miura 2017; but see Jeffries et al. 2018).

Whether this heterogametic transition involves the same chromosome pair remains to be determined. No genome assembly is yet available for bufonids, and insufficient numbers of putatively sex‐linked markers could be aligned to published references. The male‐specific karyotype dimorphism found in *B. spinosus* by Skorinov et al. (2018) was on chromosome 6 (like in *Strauchbufo radei*, Deng and Shang 1984), but this needs clarification with additional samples. The amount of XY markers flagged here (Table 1) is comparable to that of homomorphic sex chromosomes identified in other amphibians with similar datasets and methodologies (Brelsford et al. 2016b; Jeffries et al. 2018). We also found only two Y‐specific tags (Table 1), which suggests little molecular differentiation between the Y and the X. Additional analyses will be needed to ascertain the identity and degree of differentiation/degeneracy of the sex chromosomes found in *Bufo*.

Despite their different sex‐determining systems, the reproductive barriers of *B. spinosus* (XY) and *B. bufo* (ZW) are not complete. They can still produce fertile offspring and experience gene flow at range margins. In Normandy, Trujillo et al. (2017) documented an all‐hybrid population composed of reproducing males and females, some directly observed in amplexus. We detected slight peaks of linkage disequilibrium and heterozygosity in the closest contacting populations (Fig. S6), consistent with a recent origin of the introgression, as also expected given the currently overlapping distributions of the species (Arntzen et al. 2020). Yet, the narrow geographic transitions documented in southeastern France (∼10 km) and Normandy (∼20 km, Arntzen et al. 2016) suggest a dynamic of tension zones, where post‐zygotic selection against hybrids efficiently prevents the parental genomes to merge. Common toads are very mobile (Smith and Green 2005) so dispersal should be unaffected by landscape barriers: the alleles that successfully crossed the contact zone diffused far within species ranges (Fig. 2). Moreover, the transition does not follow the Rhône and the Isère rivers (as found by Arntzen et al., 2017, 2020), but was located in the woody hills north of the Isère and east of the Rhône (Fig. 2), and which offer many suitable habitats for common toads (C.D. and N.R. pers. obs.). Its present position potentially results from complex demographic processes during the Late Quaternary. The deep and asymmetric introgression at mitochondrial (on the *B. spinosus* side) and nuclear (on the *B. bufo* side) markers could indicate hybrid zone movement. van Riemsdijk et al. (2019) dissected such movements in Normandy, while Arntzen et al. (2017) interpreted the cytonuclear discordances in southeastern France as the consequences of successive range shifts since the last glaciation. This signal could also arise due to adaptive introgression (of *B. bufo* mtDNA) and/or mtDNA‐nuclear incompatibilities (with *B. spinosus* mtDNA) (Toews and Brelsford 2012), but here the geographic extent of the cytonuclear discordances remain compatible with neutral expectations (Bonnet et al. 2017). Our bioclimatic models bring indirect support to the movement hypothesis: *B. bufo* could have formerly occupied Mediterranean France (Fig. 4) but was replaced by a northeastward expansion of *B. spinosus* (which seemingly performs better in these ranges, Fig. S11), to the point that only its mtDNA remains in the south. *Bufo spinosus* may continue to locally expand further north if conditions become more suitable due to global warming. This is however specific to our particular study area: the 800‐km‐long *Bufo* transition spans heterogeneous environments from Provencal France to the British Channel, and distinct bioclimatic factors were shown to mediate the boundaries in other parts of the parapatric ranges (Arntzen et al. 2020). Beside ecology, whether phenotypic traits (acoustic or olfactive) contribute to some pre‐mating barriers also remains to be tested.

Although insufficient to fully prevent interspecies gene flow, can the different sex chromosomes of *B. bufo* and *B. spinosus* contribute to their mechanisms of post‐zygotic isolation? The dominance theory of Haldane's rule predicts lower fitness for offspring exposed to recessive sex‐linked incompatibilities in the heterogametic sex, in turn causing the large X‐effect on hybrid sterility (Schilthuizen et al. 2011). In our system, 75% of the progeny of females *B. bufo* (ZW) and males *B. spinosus* (XY) should carry a Y, a W, or both, and may thus be more sensitive than the reverse cross (XX *B. spinosus* female × ZZ *B. bufo* male) where only homogametologs (Z and X) segregate. Under these assumptions, hybrids from the *B. spinosus* matriline should have a greater fitness and thus be more abundant at the species transition. However, here we rather observed the opposite, that is, many *B. spinosus* backcrosses carrying *B. bufo* mtDNA. The dominance predictions of Haldane's rule may thus not apply to this system, perhaps because the heterogametologs are not sufficiently degenerated to bear enough recessive deleterious mutations. This was advocated to explain the absence of large X‐effects in green toads (Gerchen et al. 2018). Nevertheless, the cyto‐nuclear discordance we observed in the hybrid zone may still originate from conflicts between the genes involved in the sex determination cascade, and which could lead to differential sex‐ratio and fitness among the possible F1 hybrid genotypes (XZ, XW, YZ, YW) and those obtainable from backcrossing (YY, WW). For instance, the lack of introgression by *B. spinosus* mtDNA would fit the hypothesis that the XZ hybrids obtained from *B. spinosus* mothers (XX) and *B. bufo* fathers (ZZ) only develop as males. Finally, a role of the distinct sex chromosomes of *B. bufo* and *B. spinosus* in driving their divergence at secondary sexual traits (as in sticklebacks, Kitano et al. 2009) would be worth investigating, even though sexual dimorphism in amphibians seems controlled by the differential expression of autosomal genes rather than by sex‐linked polymorphisms (e.g., Ma et al. 2018a, 2018b; Veltsos et al. 2020).

Overall, the *B. bufo*/*spinosus* study system is somewhat reminiscent of the Japanese wrinkled frog (*Glandirana rugosa*), where closely related geographic forms bear homologous XY and ZW chromosomes and meet in secondary contacts, although without obvious incompatibilities (Miura 2007). In an extensive survey of this fascinating system, Ogata et al. (2018) experimentally produced reciprocal F1s and backcrosses, showing that YZ and XW embryos developed in males and females, respectively, while XZ and YW embryos developed into either sex. Subsequent crosses between these unusual genotypes produced WW and YY embryos. All WW individuals died after hatching, and YY individuals did develop, but fewer reached sexual maturity than expected. It will be fascinating to obtain similar information from *B. bufo* × *B. spinosus* crosses and assess whether the same effects on sex‐determination and heterogametolog lethality apply, and how here it relates to the partial reproductive isolation between these species. While it is not presently possible to test for differential sex‐linked versus autosomal introgression with our anonymous species‐diagnostic RAD tags, an anchored reference genome would allow to locate these tags and perform such test to assess whether sex‐linked genes play a disproportionate role in driving incompatibilities (large X‐ and Z‐effects). By characterizing a heterogametic transition between these hybridizing common toads, our study thus provides the first step to understand how labile sex determination mechanisms can interact with speciation processes. We call to integrate this model system in a comparative framework and address the question more generally, notably by targeting other pairs of parapatric lineages that carry different sex chromosomes, as increasingly reported in anuran amphibians (e.g., *Hyla orientalis*/*savignyi* in Turkey, Dufresnes et al. 2015, 2020; *Rana japonica* in Japan, *R. pipiens* in the United States, Jeffries et al. 2018).

## AUTHOR CONTRIBUTIONS

C.D., N.P., and P.A.C. designed the study. C.D., N.R., and P.A.C. conducted the field work. C.D., D.L.J., S.N.L., and B.R.K. performed the analyses. C.D. and D.L.J. wrote the article.

## DATA ARCHIVING

The mitochondrial barcoding data are available in Table S1. The RAD‐seq (raw sequence reads) were uploaded on the NCBI sequence read archive (SRA) under BioProject PRJNA542138.

Associate Editor: J. Mank

## Supporting information


**Fig. S1**: Anatomical examination of *B. bufo* gonads.
**Fig. S2**: Occurrence records used to build the species distribution models.
**Fig. S3**: Parameter testing for identification of sex‐linked markers with SLM finder.
**Fig. S4**: PCA of *Bufo* allele frequency in southeastern France.
**Fig. S5**: Correlation between STRUCTURE and PCA scores.
**Fig. S6**: Population averages of ancestry, heterozygosity and linkage disequilibrium indices along the hybrid zone transect.
**Fig. S7**: Triangle plot of individual heterozygosity *vs* nuclear ancestry.
**Fig. S8**: Distribution of the cline parameters.
**Fig. S9**: Comparison of the tail parameters of the clines between the *B. bufo* and the *B. spinosus* side.
**Fig. S10**: Multivariate analyses of environmental conditions at *B. bufo* and *B. spinosus* occurrence records.
**Fig. S11**: Relationship between species occurrence probability and distance along our transect in southeastern France.

**Table S1**: Details on the samples included this study.

**Table S2**: Parameter sets tested in SLM Finder.

**Table S3**: Performance metrics and variable contributions of the species distribution models.
