# Supplementary material for: Hybridization and introgression between toads with different sex chromosome systems
Source: Evol Lett. 2020 Aug 19;4(5):444–56. doi: 10.1002/evl3.191 (PMC7523563; doi:10.1002/evl3.191)
Supplement: Supplementary file 1 — Fig. S1: Anatomical examination of B. bufo gonads. Fig. S2: Occurrence records used to build the species distribution models. Fig. S3: Parameter testing for identification of sex‐linked markers with SLM finder. Fig. S4: PCA of Bufo allele frequency in southeastern France. Fig. S5: Correlation between STRUCTURE and PCA scores. Fig. S6: Population averages of ancestry, heterozygosity and linkage disequilibrium indices along the hybrid zone transect. Fig. S7: Triangle plot of individual heterozygosity vs nuclear ancestry. Fig. S8: Distribution of the cline parameters. Fig. S9: Comparison of the tail parameters of the clines between the B. bufo and the B. spinosus side. Fig. S10: Multivariate analyses of environmental conditions at B. bufo and B. spinosus occurrence records. Fig. S11: Relationship between species occurrence probability and distance along our transect in southeastern France. [file EVL3-4-444-s001.pdf]

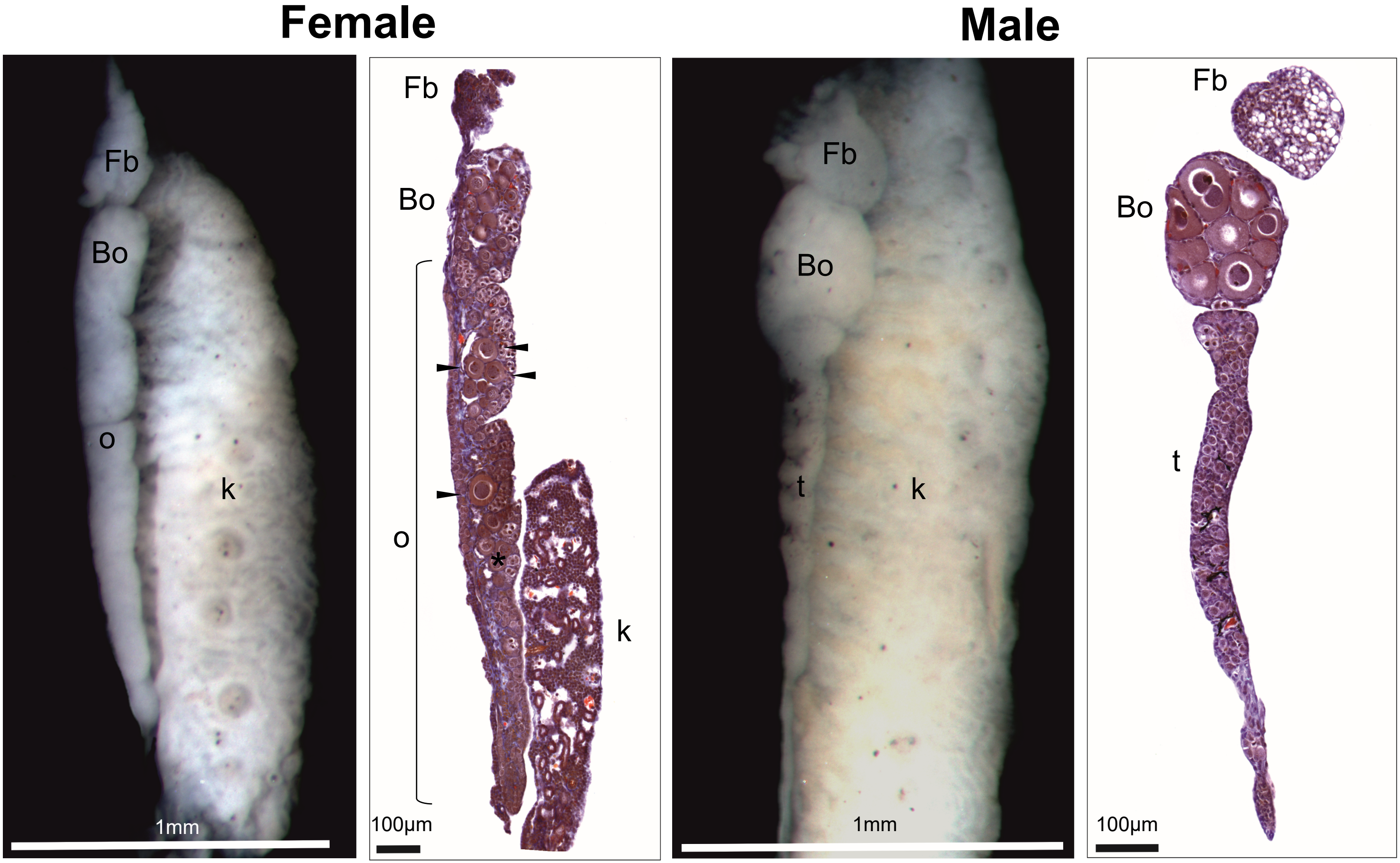

Fig S1: Histological sections of gonads for the identification of the phenotypic sex of metamorphing *B. bufo* individuals. Left gonads of a female (left) and a male (right) are illustrated, respectively. k: kydney; Fb: fat body; Bo: bidders ordan; o: ovary; t: testis. In the female ovary, the asterisk highlights a forming ovarian cavity and the arrows point to diplotene oocytes.

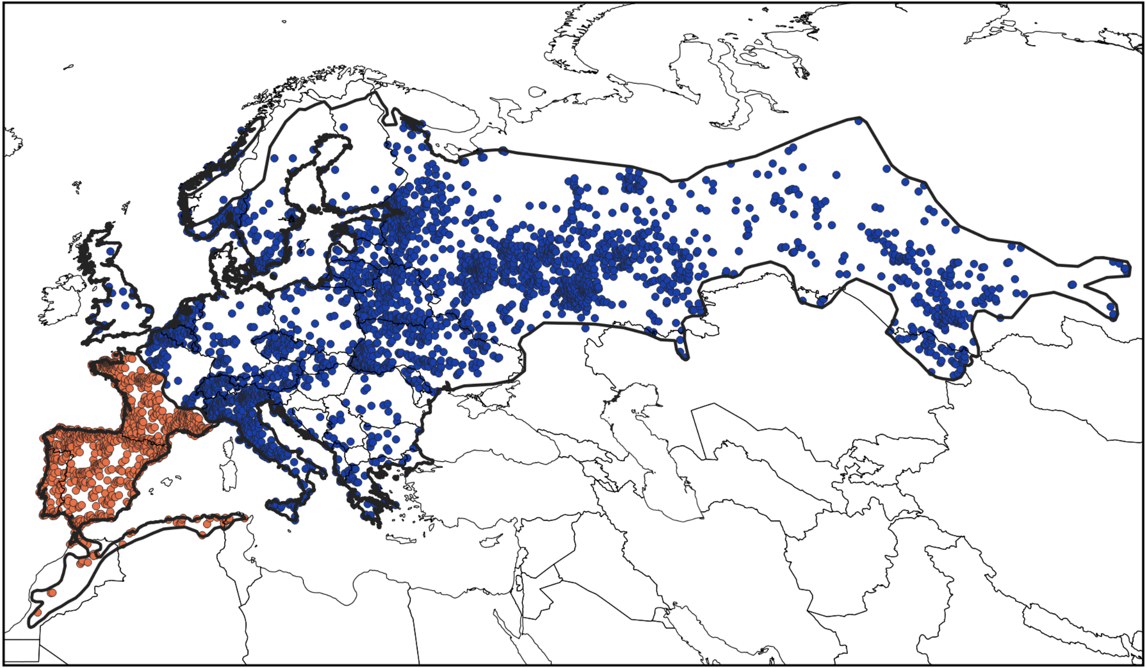

Fig. S2: Occurence records of *B. bufo* (blue) and *B. spinosus* (red) used to build the bioclimatic models

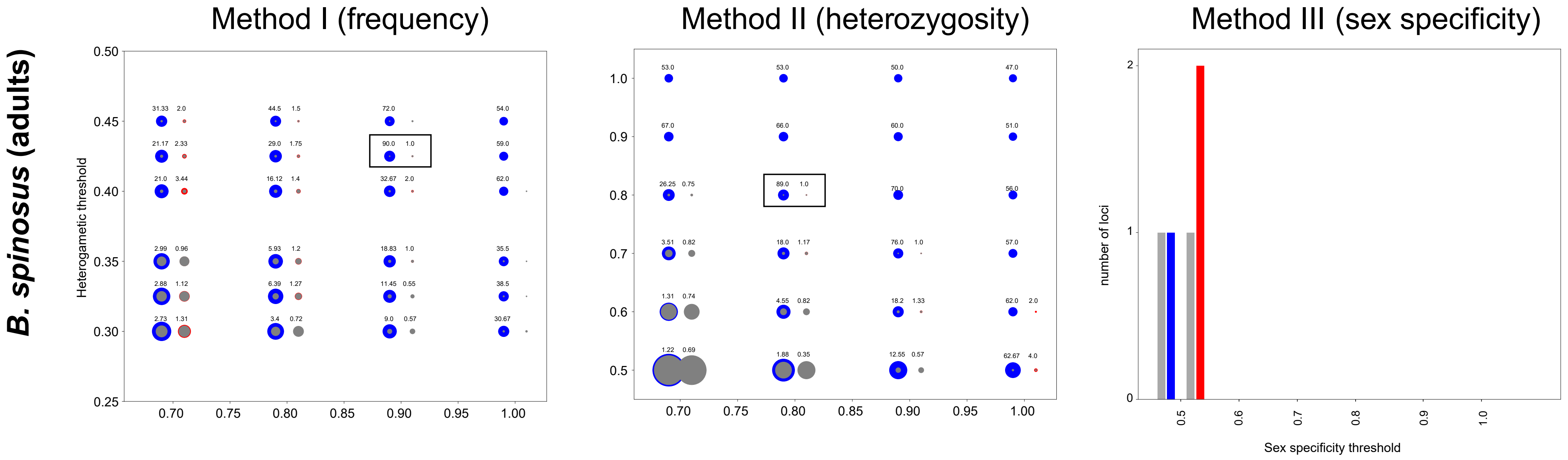

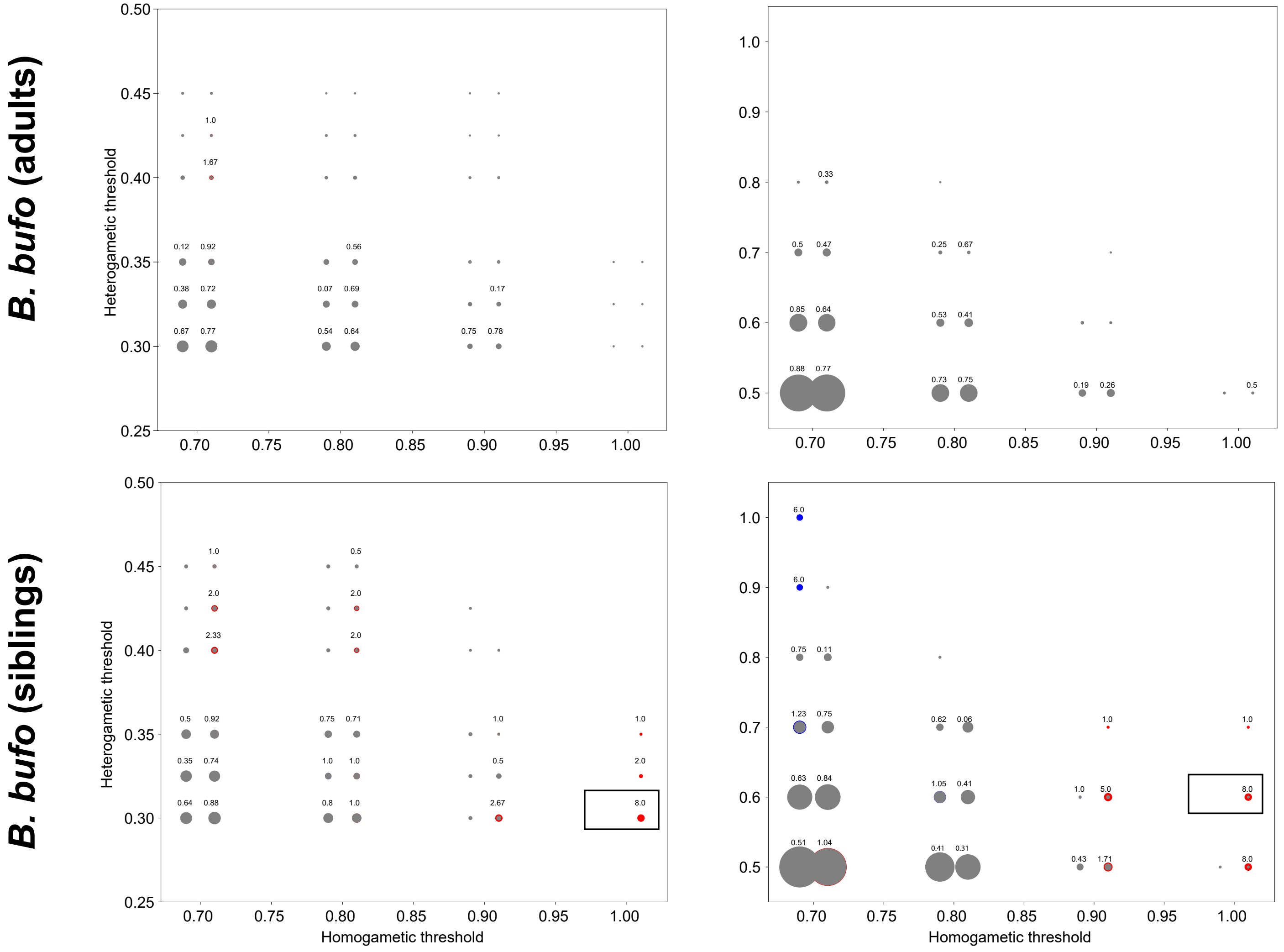

Fig. S3: Parameter testing for identification of sex linked markers with each method of SLM Finder from the *B. spinosus* adult dataset (top), the *B. bufo* adult dataset (middle) and the *B. bufo* sibling dataset (bottom). For each combination of homogametic and heterogametic threshold parameters, the number of markers in support of either XY (left) or ZW (right) is plotted by proportional circles. Grey circles represent the 95th percentile for the distribution of markers identified in permutation tests (n = 100), while the overlayed colored circles display the observed data (i. e. number of markers in the real sex assignments). If the latter are not visible, then the parameter combination did not result in reliable support for sex linkage of any markers (e. g. *B. bufo* adults). Associated values are the ratio of sex-linked markers obtained from the observed data (real sex assignments) over the null distribution (95th percentile of the permutations). The larger this number, the fewer false positives are expected relative to the number of truly sex linked markers. When relevant, this number was used to identify the optimal parameters to use for the final analysis of each dataset (black frames). For method III, which only detected sex-linked markers in the *B. spinosus* adult dataset, their number in respect to the six parameters tested is plotted.

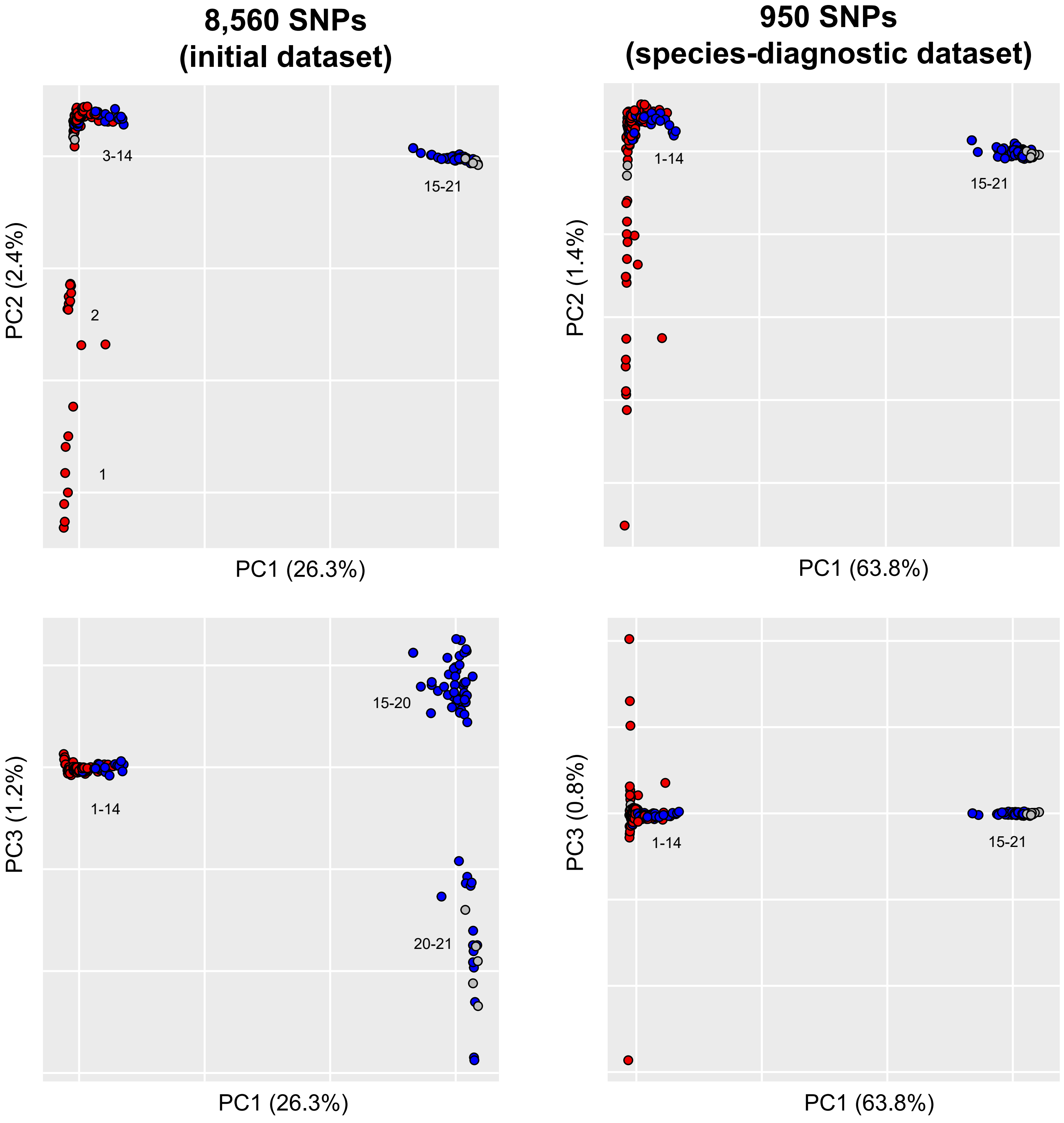

Fig S4: PCA on individual allele frequency from the initial and species-diagnostic datasets. Each dot represents an individual, colored by its mtDNA (blue: *B. bufo*; red: *B. spinosus*; grey: NA). Numbers indicate locality codes (as in Fig. 2 and Table 1).

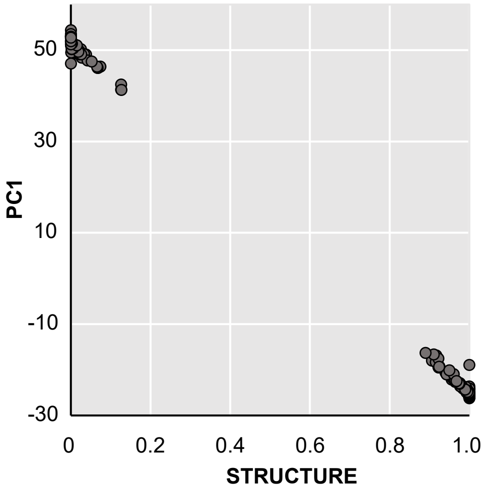

Fig S5: Correlation between the ancestry of *B. spinosus* and the first PCA component (PC1).

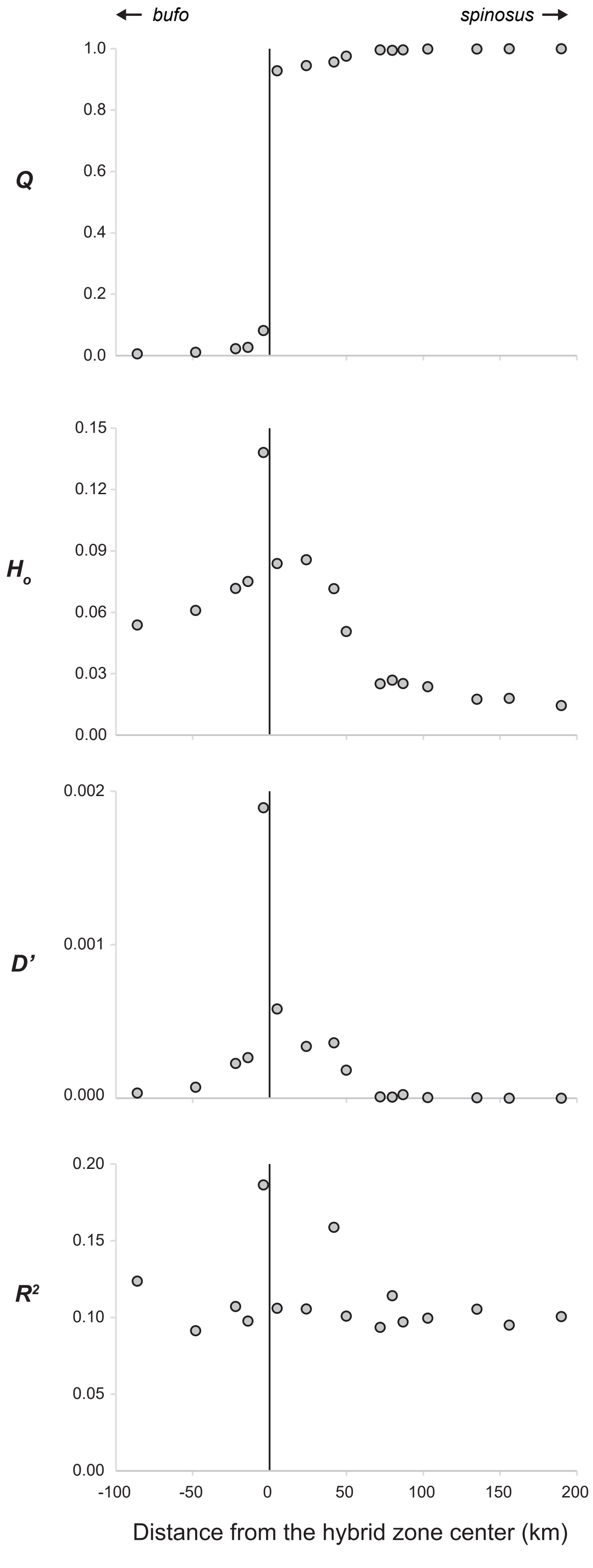

Fig. S6: Mean ancestry *Q* (*B. spinosus*), observed heterozygosity (H_o), admixture linkage disequilibrium (*D’*) and linkage disequilibrium averaged over pairs of loci (correlation coefficient R^2) computed from 950 species-diagnostic SNPs in 16 populations sampled along our *Bufo* transect in southwestern France (edge populations used for species-diagnostic SNPs filtering are not included). Geographic positions along the transect are displayed as the deviation from the median cline center (thick lines).

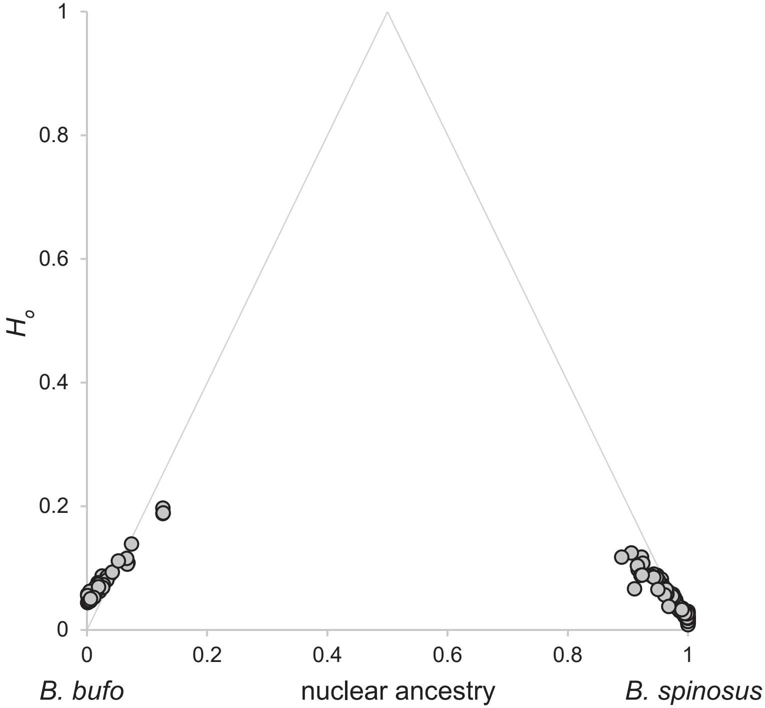

Fig. S7: Triangle plot of individual nuclear ancestry (of *B. spinosus*) at 950 species-diagnostic SNPs *vs* observed heterozygosity (H_o) in the *B. bufo* / *spinosus* hybrid zone.

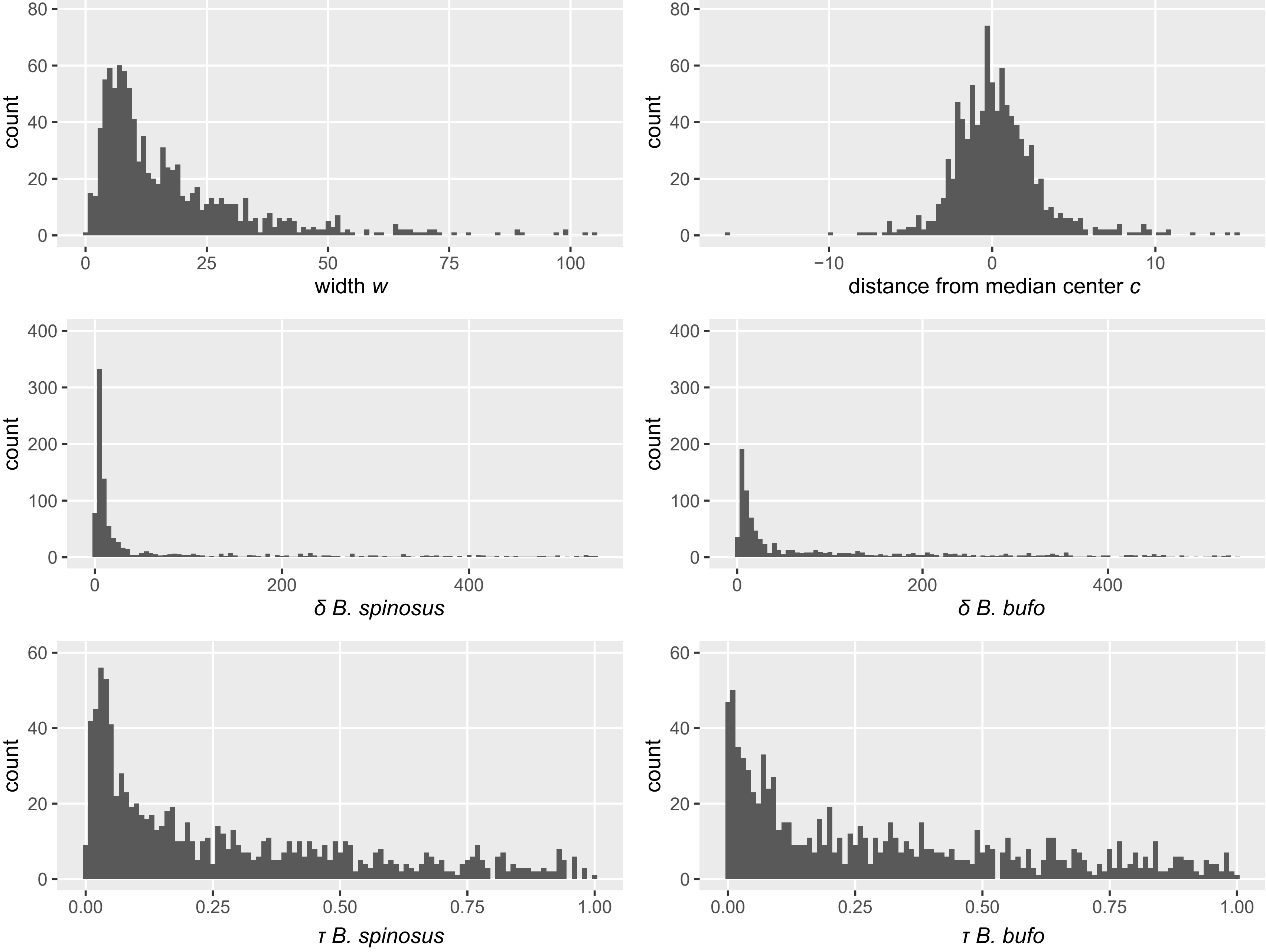

Fig S8: Distribution of the six cline parameters estimated for each of the 950 species-diagnostic loci. *w*: cline width; *c*: cline center; *δ*: length of the exponential tails; *τ*: slope of the exponential tails.

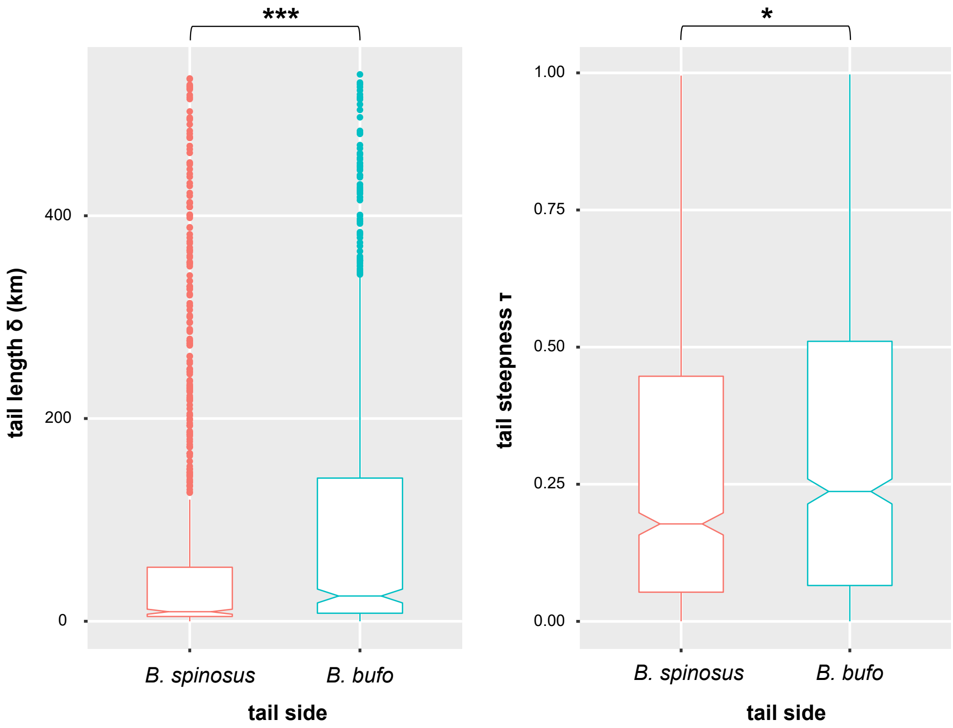

Fig S9: Comparisons of the tail parameters from the 950 species-diagnostic clines. Introgression tails are significantly longer (higher δ) and steeper (higher τ) on the northeastern (*B. bufo*) side than the southwestern (*B. spinosus*) side of the transect (Wilcoxon signed rank tests).

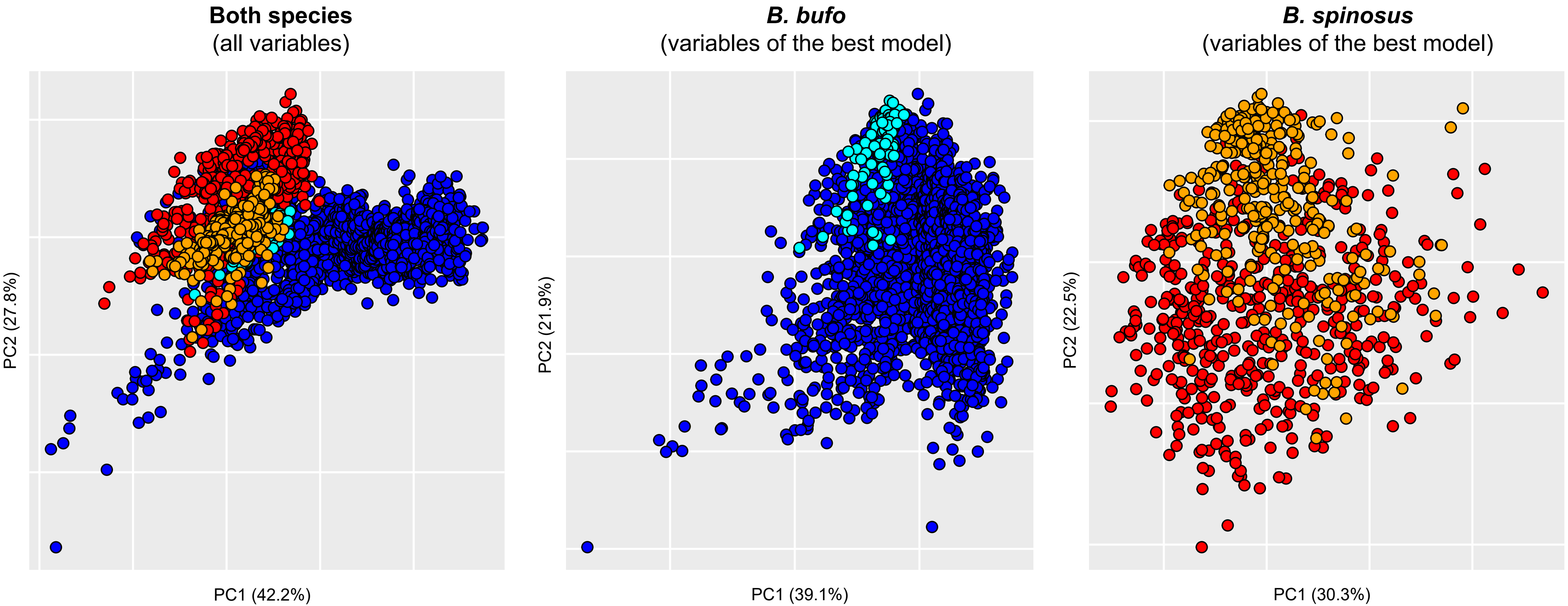

Fig S10: Multivariate analyses (PCA) of environmental conditions at *B. bufo* and *B. spinosus* localities in parental (blue and red, respectively) and french parapatric ranges (orange and cyan, respectively). The left PCA is built from all available variables; the central and right panels are built only from the variables retained in the ecological model selected for each species (see Table S2)

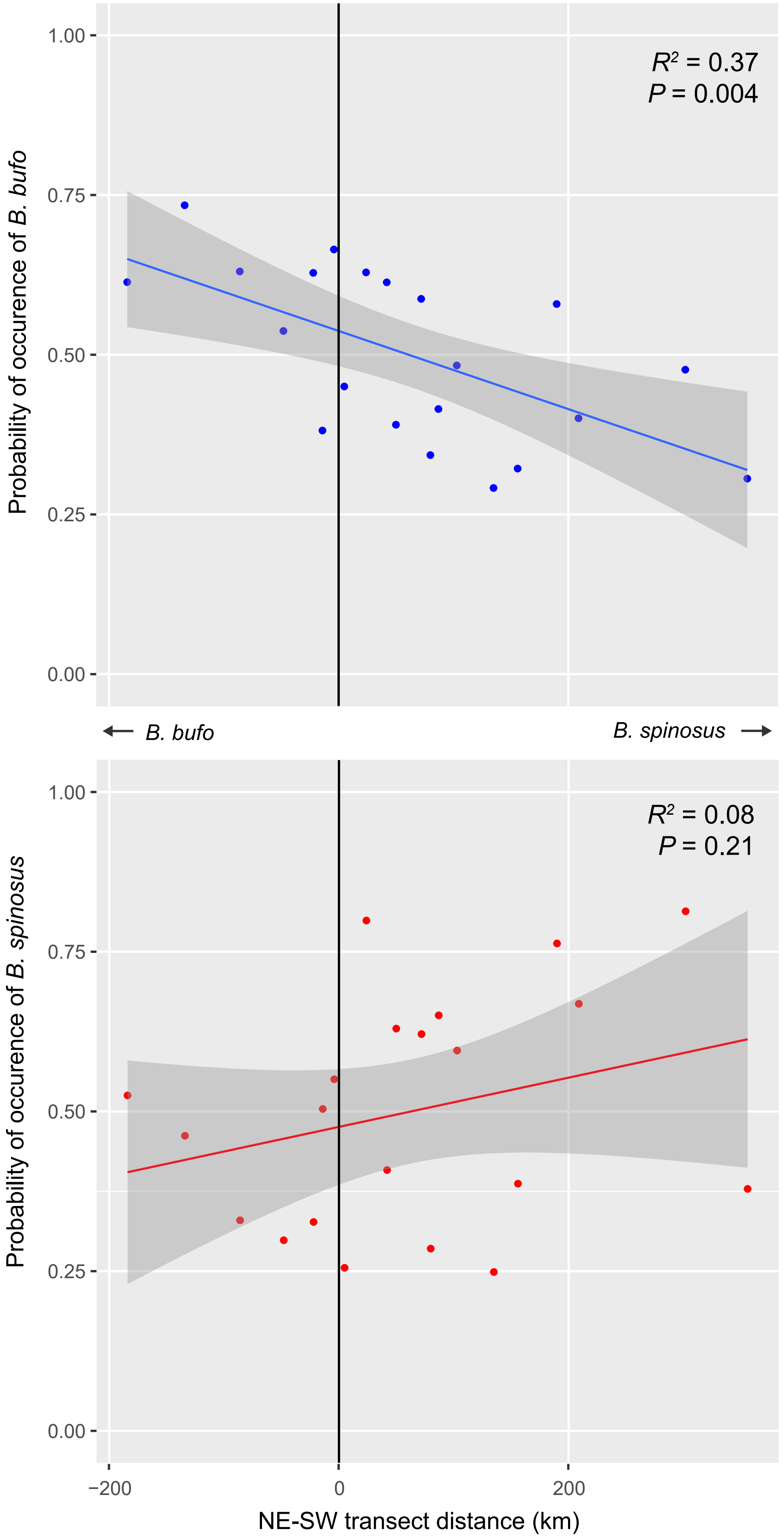

Fig. S11: Probability of occurence of *B. bufo* (top) and *B. spinosus* (bottom) along our transect in southeastern France. The suitability significantly changes with geographic distances in *B. bufo* but not in *B. spinosus* (linear regression)
