# Supplementary material for: Hybridization and introgression between toads with different sex chromosome systems
Source: Evol Lett. 2020 Aug 19;4(5):444–56. doi: 10.1002/evl3.191 (PMC7523563; doi:10.1002/evl3.191)
Supplement: Supplementary file 3 — Table S2: Parameter sets tested in SLM Finder. [file EVL3-4-444-s003.docx]

**Table S2:** Parameter sets used to find sex-linked markers with the three methods of SLM Finder. Optimal parameter sets were I-17, II-10, III-1 for the *B. spinosus* adult dataset, and I-19, II-20 for the *B. bufo* sibling dataset (see Fig. S3).

| **Set** | **Homogametic threshold** | | **Heterogametic threshold** |  | **Set** | **Homogametic threshold** | **Heterogametic threshold** |  | **Set** | **Sex specificity threshold** |
| --- | --- | --- | --- | --- | --- | --- | --- | --- | --- | --- |
|  |  | |  |  |  |  |  |  |  |  |
| ***Method I*** | |  |  |  | ***Method II*** |  |  |  | ***Method III*** |  |
| I-1 | 0.7 | | 0.3 |  | II-1 | 0.7 | 0.5 |  | III-1 | 0.5 |
| I-2 | 0.7 | | 0.325 |  | II-2 | 0.7 | 0.6 |  | III-2 | 0.6 |
| I-3 | 0.7 | | 0.35 |  | II-3 | 0.7 | 0.7 |  | III-3 | 0.7 |
| I-4 | 0.7 | | 0.4 |  | II-4 | 0.7 | 0.8 |  | III-4 | 0.8 |
| I-5 | 0.7 | | 0.425 |  | II-5 | 0.7 | 0.9 |  | III-5 | 0.9 |
| I-6 | 0.7 | | 0.45 |  | II-6 | 0.7 | 1 |  | III-6 | 1 |
| I-7 | 0.8 | | 0.3 |  | II-7 | 0.8 | 0.5 |  |  |  |
| I-8 | 0.8 | | 0.325 |  | II-8 | 0.8 | 0.6 |  |  |  |
| I-9 | 0.8 | | 0.35 |  | II-9 | 0.8 | 0.7 |  |  |  |
| I-10 | 0.8 | | 0.4 |  | II-10 | 0.8 | 0.8 |  |  |  |
| I-11 | 0.8 | | 0.425 |  | II-11 | 0.8 | 0.9 |  |  |  |
| I-12 | 0.8 | | 0.45 |  | II-12 | 0.8 | 1 |  |  |  |
| I-13 | 0.9 | | 0.3 |  | II-13 | 0.9 | 0.5 |  |  |  |
| I-14 | 0.9 | | 0.325 |  | II-14 | 0.9 | 0.6 |  |  |  |
| I-15 | 0.9 | | 0.35 |  | II-15 | 0.9 | 0.7 |  |  |  |
| I-16 | 0.9 | | 0.4 |  | II-16 | 0.9 | 0.8 |  |  |  |
| I-17 | 0.9 | | 0.425 |  | II-17 | 0.9 | 0.9 |  |  |  |
| I-18 | 0.9 | | 0.45 |  | II-18 | 0.9 | 1 |  |  |  |
| I-19 | 1 | | 0.3 |  | II-19 | 1 | 0.5 |  |  |  |
| I-20 | 1 | | 0.325 |  | II-20 | 1 | 0.6 |  |  |  |
| I-21 | 1 | | 0.35 |  | II-21 | 1 | 0.7 |  |  |  |
| I-22 | 1 | | 0.4 |  | II-22 | 1 | 0.8 |  |  |  |
| I-23 | 1 | | 0.425 |  | II-23 | 1 | 0.9 |  |  |  |
| I-24 | 1 | | 0.45 |  | II-24 | 1 | 1 |  |  |  |
|  |  | |  |  |  |  |  |  |  |  |
