# Supplementary material for: Hybridization and introgression between toads with different sex chromosome systems
Source: Evol Lett. 2020 Aug 19;4(5):444–56. doi: 10.1002/evl3.191 (PMC7523563; doi:10.1002/evl3.191)
Supplement: Supplementary file 4 — Table S3: Performance metrics and variable contributions of the species distribution models. [file EVL3-4-444-s004.docx]

**Table S3:** Performance metrics for the final models for *Bufo bufo* and *B. spinosus*, and relative contribution (%) of variables.

| **Variable** | ***B. bufo*** | ***B. spinosus*** |
| --- | --- | --- |
| Partial ROC | 0 | 0 |
| Omission rate 5% | 0.05 | 0.05 |
| AICc | 105077 | 24513 |
| Delta AICc | 0 | 0 |
| Regularization parameter | 0.5 | 2 |
| Response type of feature classes | linear | quadratic |
| Annual mean temperature (Bio1 ) | 0.8 | - |
| Mean diurnal range (Bio 2) | 16.4 | - |
| Isothermality (Bio 3) | 1.5 | 1.6 |
| Minimum temperature of coldest month (Bio 6) | - | 0.1 |
| Temperature annual range (Bio 7) | 1.6 | - |
| Mean temperature of wettest quarter (Bio 8) | 2.6 | 11.7 |
| Precipitation of driest month (Bio 14) | 1.0 | 0 |
| Precipitation seasonality (Bio 15) | 2.5 | - |
| Precipitation of wettest quarter (Bio 16) | 0.6 | 0.2 |
| Precipitation of warmest quarter (Bio 18) | 3.0 | - |
| Precipitation of coldest quarter (Bio 19) | 0 | - |
| Broadleaf forest | 15.9 | 7.3 |
| Needleleaf forest | 1.1 | 2.5 |
| Mixed forest | 1.8 | 12.9 |
| Shrubs | 12.9 | 3.0 |
| Barren | 0.8 | 2.4 |
| Herbaceous vegetation | 6.2 | 0.7 |
| Cultivated vegetation | 14.8 | 38.3 |
| Altitude | 0.7 | 2.5 |
| Aridity index | 0.3 | 0 |
| Aspect | 0 | 0.1 |
| Exposition | 0 | 0 |
| Habitat homogeneity | 0.1 | 0 |
| Slope | 0.1 | 0 |
| Terrain roughness index | 0.3 | 0 |
| Tree coverage percent | 14.8 | 16.6 |
